# Misdiagnosis of rarest subtype of sporadic Creutzfeldt Jakob Disease: a case report

**DOI:** 10.1186/s12883-023-03318-z

**Published:** 2023-07-18

**Authors:** Aemal Aziz Jabarkhil, Aziz Rahman Rasib, Abdullah Asady, Farhad Farzam

**Affiliations:** 1 Department of Neuropsychiatry, Kabul University of Medical Sciences, Kabul, 1001 Afghanistan; 2 Department of Microbiology, Kabul University of Medical Sciences, Kabul, 1001 Afghanistan; 3 Department of Medical Imaging and Radiation Sciences, Kabul University of Medical Sciences, Kabul, 1001 Afghanistan

**Keywords:** Sporadic Creutzfeldt-Jakob disease, VV1 subtype, Magnetic resonance imaging, Case report

## Abstract

**Background:**

Creutzfeldt–Jakob disease (CJD), is a deadly degenerative condition of the central nervous system marked by rapidly progressive dementia. Magnetic resonance imaging (MRI) abnormalities in the cerebral cortex, basal ganglia, thalamus, and cerebellum could indicate severe acute diseases caused by a variety of factors. Although their MRI patterns may resemble those of CJD, clinical history, additional MRI findings, and laboratory testing are all necessary to provide a reliable difference. Here, we report a misdiagnosed case of probable VV1 subtype of sporadic CJD (sCJD) in which follow-up MRI supported the diagnosis.

**Case presentation:**

A 41-year-old male patient attended the Neuropsychiatry Department with rapidly progressive dementia, akinetic mutism, and difficulty walking and speaking. His problem began with forgetfulness, disorganized behavior, and disorganized speech 7 months earlier which progressed rapidly and was accompanied by aphasia, apraxia, agnosia, and akinetic mutism in the last 2 months. On neurologic examination, hypertonia, hyperreflexia, frontal ataxia, bradykinesia, gait apraxia, and aphasia were noted. Based on clinical features and rapid symptoms progression the likely diagnosis of CJD was suspected. MRI and electroencephalography (EEG) were advised. MRI revealed features of diffuse cortical injury of both cerebral hemispheres also involving bilateral corpus striatum with evidence of cerebral volume loss. EEG showed lateralized periodic theta slow waves on the right side. According to the CDC’s diagnostic criteria for CJD, the diagnosis of probable sCJD was established. Supportive care and symptomatic treatment are provided for the patient. After a 1-month follow up the patient’s condition deteriorated significantly. The time-lapse from the first reported symptom to death was about 13 months.

**Conclusion:**

The need of addressing CJD in patients presenting with rapidly progressive dementia is highlighted in this case report. In the early stages of the disease, interpretation of MRI results might cause diagnostic difficulties; therefore, follow-up MRI is critical in obtaining the correct diagnosis.

## Background

Creutzfeldt–Jakob disease (CJD) is a lethal degenerative disease of the central nervous system determined by gravely progressive dementia. It is subdivided into four subtypes: sporadic (sCJD), familial (fCJD), variant (vCJD), and iatrogenic (iCJD). The annual global incidence of CJD is 1 to 2 cases per 1 million population, and sCJD accounts for the majority (approximately 85% of CJD cases) [1]. The distinctive clinical picture includes intellectual disability, myoclonus, visual or cerebellar abnormalities, pyramidal or extrapyramidal signs, and akinetic mutism. Due to a variety of factors, including variable presentations and a lack of appropriate gold-standard diagnostic methods in the clinical context, it is underdiagnosed and increasingly misdiagnosed. The condition has a poor prognosis, with a life expectancy ranging from a few weeks to a year. Furthermore, there is no specific medication available to decrease the disease’s progression [2].

The use of follow-up Magnetic resonance imaging (MRI) for CJD diagnosis has not been widely reported. Here, we report a case of probable VV1 subtype of sCJD misdiagnosed as herpes encephalitis, schizophrenia, and frontotemporal dementia, follow-up MRI supported in the establishment of diagnosis.

### Case presentation

A 41-year-old male patient attended the Neuropsychiatry Department of Ali Abad Teaching Hospital, Kabul, Afghanistan with rapidly progressive dementia, akinetic mutism, and difficulty walking and speaking. Due to his difficulty speaking and mutism, he was unable to provide additional information. Per his son, his problem began with forgetfulness, disorganized behavior, and disorganized speech 7 months earlier which progressed rapidly and was accompanied by aphasia, apraxia, agnosia, and akinetic mutism in the last 2 months. Throughout this period, he was admitted to different governmental and private hospitals under different conditions, including herpes encephalitis, schizophrenia, and frontotemporal dementia, and had received several medicines but his condition deteriorated quickly day by day. Nothing significant was found in his past medical, drug, and family history. In the years before the onset of the current illness, he had not had any operations including eye operations or stitching of wounds. He had not received an organ or tissue transplant, including a corneal or bone marrow transplant. He had not received a blood transfusion (blood components or plasma products), transfusion of albumin, or immunoglobulin. No blood relatives of the patient died with dementia or are alive with dementia. He had no history of traveling outside of Afghanistan.

On general physical examination, the patient seemed awake and likely aware. His vital signs were normal, and no abnormality was found in the examination of the cardiovascular, respiratory, gastrointestinal, and genitourinary systems. On neurologic examination, hypertonia, hyperreflexia, frontal ataxia, bradykinesia, gait apraxia, and aphasia were noted. Routine lab investigations including full blood count, fasting plasma glucose, serum electrolytes, liver function test, renal function test Vitamin B12 level, and thyroid function test were within normal ranges. Cerebrospinal fluid (CSF) analysis showed normal cells, protein, and glucose.

**Fig. 1:**
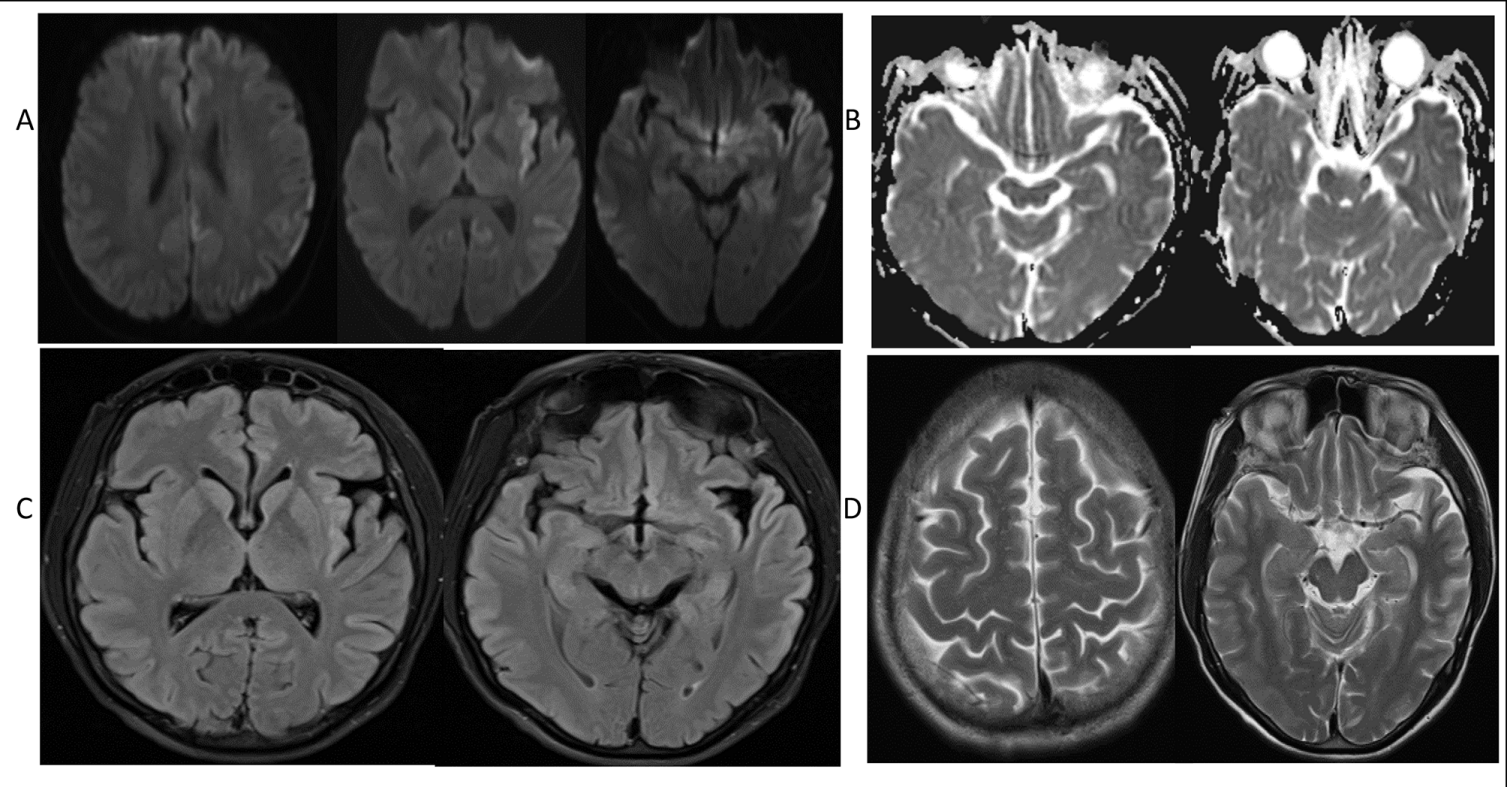
Diffusion Weighted Images (DWI); diffuse cortical injury evident by diffusion restriction also involving the corpus striatum(1 **A**); Susceptibility Weighted Images (SWI); diffuse cortical injury evident by diffusion restriction(1**B**); FLAIR; Diffuse cortical hyperintensity(1 **C**); T2 WI, evidence of mild volume loss in both cerebral hemispheres(1**D**)

A report of the MRI which was done 6 months before when he was suspected of suffering from herpes simplex encephalitis showed mild volume loss, flair high signal and patchy diffusion restriction in bilateral frontotemporal lobes. Based on clinical features and rapid symptoms progression the likely diagnosis of CJD was suspected. Follow-up MRI and electroencephalography (EEG) were advised. Follow-up MRI revealed features of diffuse cortical injury of both cerebral hemispheres evident by diffusion restriction also involving bilateral corpus striatum on Diffusion Weighted Imaging (DWI) (Fig. 1A); diffuse cortical injury evident by diffusion restriction on Susceptibility Weighted Imaging (SWI) (Fig. 1B); Diffuse cortical hyperintensity on Fluid-attenuated inversion recovery (FLAIR) (Fig. 1C); with evidence of cerebral volume loss on T2 WI (Fig. 1D). EEG showed lateralized periodic theta slow waves on the right side.

According to the centers for disease control and prevention (CDC), diagnostic criteria for CJD [3], and the diagnosis of probable sporadic CJD was established. Supportive care and the following symptomatic treatment protocol were planned: donepezil 5 mg initially and increased up to 10 mg gradually; memantine 5 mg initially and increased up to 20 mg gradually; quetiapine 25 mg daily. After a 1-month follow up the patient’s condition deteriorated significantly and become completely bedridden and incontinent. The patient died after an illness duration of approximately 13 months.

## Discussion and conclusion

This case was diagnosed with probable sCJD based on the CDC criteria for the disease [3]. The key findings included rapidly progressive dementia, akinetic mutism, pyramidal/extrapyramidal signs (hyperreflexia, frontal ataxia, gait apraxia, aphasia, hypertonia, and bradykinesia), diffuse cortical injury of both cerebral hemispheres evident by diffusion restriction also involving bilateral corpus striatum on DWI, and exclusion of alternative diagnosis by detailed clinical history, routine lab investigations, CSF analysis, and neuroimaging. To our knowledge, this is the first case of probable sCJD reported from Afghanistan.

sCJD is classified into seven subtypes, with the VV1 subtype being the rarest, with a frequency of roughly 1%. The majority of the patients who have been reported are young males. Psychiatric or cognitive impairments are usually the first signs of the condition. Extrapyramidal signs, ataxia, and myoclonus appear later in the course. The disease in these individuals mostly affects the corticostriatal areas, while other subcortical structures, such as the cerebellum, remain relatively unaffected. In the cerebral cortex, despite the significant spongiform alteration, prion protein(PrP)immunohistochemistry reveals a slight punctuate staining [4]. The classic CJD triad of dementia, myoclonus, and PSWCs on EEG is a prevalent and early manifestation in MM1 and MV1 individuals. Moreover, ocular signs may precede severe dementia in nearly 30% of patients. Ataxia upon onset, isolated or in conjunction with mild cognitive decline is a solid characteristic of VV2 individuals. Contrary to the MM1 and MV1 individuals, the VV2 individuals generally do not show PSWCs on EEG, and around a third of them do not have obvious myoclonus. Nocturnal psychomotor agitation and insomnia, and lack of PSWCs on EEG are classic clinical characteristics of the MM2-thalamic phenotype. In the MM2-cortical phenotype, dementia is the major sign, and ocular or cerebellar signs, and PSWCs on EEG are typically not present. The VV1 individuals, such as MM2-cortical individuals, exhibit a clinical phenotype with predominant cortical signs and progressive dementia, with neither characteristic EEGs nor early cerebellar signs. Though, pathological features like the type of spongiform changes, and the pattern of PrP deposition, distinctly differentiate the two groups [5].

Although a definite diagnosis of sCJD subtypes requires prion protein gene analysis, prion protein western blotting, and neuropathological examination. Due to the lack of feasibility of these tests in Afghanistan, they were not performed. However, based on clinical-imaging findings (young male, the appearance of forgetfulness, disorganized behavior, and disorganized speech at an early stage. The appearance of bradykinesia and rigidity later in the course. corticostriatal involvement with intact cerebellum on imaging and lack of PSWCs on EEG) our patient probably is suffering from the VV1 subtype of sCJD.

CJD in young age individuals is extremely infrequent and generally the result of a genetic mutation or exogenous exposure [6]. Lack of family history of dementia and the absence of known acquired prion disease risk factors, in this case, can exclude fCJD and iCJD.

The diagnosis of sCJD takes a long time. sCJD should be considered in the differential diagnosis of patients with a rapidly progressive neurocognitive impairment who have a suspected neurodegenerative, autoimmune, viral, or toxic/metabolic etiology. Approaching differential diagnoses based on MRI could help prevent CJD misdiagnosis due to misinterpretation of imaging characteristics. As a result, it is critical to recognize common imaging findings and, more importantly, to be aware of atypical findings. MRI abnormalities in the cerebral cortex, basal ganglia, thalamus, and cerebellum could indicate severe acute diseases caused by a variety of factors. Although their MRI patterns may resemble those of sCJD, clinical history, additional MRI findings, and laboratory exams are all necessary to make a solid difference. Improved training in sCJD diagnosis is required for primary care providers and neurologists [7, 8].

Herpes simplex encephalitis presents as an acute altered mental state and a wide range of unspecific manifestations of focal encephalopathy, comprising fever, headache, neck stiffness, and personality disorder. In adults, it frequently affects the orbital frontal lobes, and the anterior and medial perspective of the temporal lobe, often asymmetrically. The main distinguishing features from sCJD are the acute febrile illness and the predominately mesial temporal lobe involvement [9]. In this case, we find several pitfalls in the diagnosis of sCJD, including; a lack of experience in diagnosing CJD due to the low incidence and prevalence of the disease, atypical clinical manifestations and imaging findings in the early stages of the disease, and ignoring the importance of serial MRI within the disease course for reaching the correct diagnosis. The clues to consider the probable diagnosis of sCJD were the lack of CSF pleocytosis, the rapid deterioration of the patient’s condition despite receiving acyclovir, and the progression of the MRI abnormalities in the bilateral corpus striatum. Patients with frontotemporal dementia can mimic sCJD by exhibiting extrapyramidal signs like rigidity, tremor, or akinesia. On MRI, frontal and/or temporal lobe atrophy is typically visible [10]. Frontotemporal dementia often has an insidious onset, progressing very gradually, and lasts longer than five years overall [11]. In this patient, overlapping symptomatology and MRI pattern of FTD with CJD in the early stages of the disease misled the diagnosis. However, the time course and the subsequent MRI pattern were more consistent with the diagnosis of sCJD. As sCJD might manifest with psychiatric symptoms, it may initially be misdiagnosed as psychosis [12]. The rapid decline in cognition with transient psychiatric symptoms, the presence of neurological signs, and typical neuroimaging findings may aid in differentiating sCJD from psychotic disorders.

We can assume that the disease is misdiagnosed in Afghanistan due to a lack of experience in diagnosing CJD, a reluctance to do a follow-up MRI due to financial constraints, and a lack of feasibility of supportive tests such as Protein misfolding cyclic amplification (PMCA), CSF analysis for 14-3-3, total tau, and real-time quaking-induced conversion (RT-QuIC).

Several pathologies present as hyperintense lesions located in the cerebral cortex on diffusion-weighted imaging. Aside from herpes simplex encephalitis and hypoglycemia, such lesions are also found in hypoxic-ischemic encephalopathy, hyperammonemia, epileptic seizures, limbic encephalitis, mitochondrial disease, intravascular lymphoma, and cerebral infarction [13].

In this case, many of these etiologies can be excluded fairly quickly with the patient’s history, normal lab investigations, lack of CSF pleocytosis, and progression of MRI changes in follow-up imaging.

The leading cause of hypoxic-ischemic encephalopathy is circulatory or respiratory failure owing to cardiac arrest, drowning, or suffocation. Hypoglycemia characteristically affects diabetics who unintentionally overuse insulin or oral antidiabetic agents also patients with an unknown insulinoma or other serious medical conditions that cause excessive glucose utilization. A review of the biochemical results and the clinical context both help in diagnosis. The main cause of hyperammonemia is an acute hepatic failure in critically ill patients. Although there may be some overlap between the MR imaging findings of sCJD and those of mitochondrial diseases, the clinical presentations will typically be distinct. The amygdala and hippocampus, two brain regions not frequently damaged in sCJD, are typically involved in limbic encephalitis [8]. Repeating the imaging scan can help distinguish between restrictive diffusion caused by a stroke and seizure, as DWI abnormality associated with seizures often disappear once the seizures are under control while those associated with stroke linger [13]. Intravascular lymphoma is an infrequent condition. It causes seizures, upper motor neuron symptoms, rapid cognitive impairment, and peripheral symptoms like neuropathy. Imaging results are consistent with those of primary CNS vasculitis [14].

. The first in vivo test to support the sCJD clinical diagnosis is the EEG. The EEG may be normal in the early stages of the illness or it may show undefined changes like delta or theta activity. Periodic lateralized epileptiform discharges (PLED) or frontal intermittent rhythmic delta activity (FIRDA) may occasionally be the first EEG finding [15].

Because there is no effective treatment for CJD, it is crucial to identify risk factors and slow disease progression. CJD has no cure, and there are no medications that can be used to manage it or track its progression. To better identify symptoms and provide effective therapies, further research is needed. Some medicines, such as clonazepam, can aid with symptomatic treatment, such as in the case of myoclonus [16].

This case report highlights the importance of considering CJD in patients presenting with rapidly progressive dementia. In Afghanistan and other developing countries, the absence of advanced diagnostic methods combined with a lack of awareness of CJD may result in a missed diagnosis. In the early stages of the disease, interpreting MRI findings can be difficult, so follow-up MRI is important for getting the appropriate diagnosis.

## Data Availability

All data generated or analyzed during this study are included in this published article.

## Abbreviations

CJD: Creutzfeldt–Jakob disease
sCJD: Sporadic Creutzfeldt–Jakob disease
iCJD: Iatrogenic Creutzfeldt–Jakob disease
fCJD: Familial Creutzfeldt–Jakob disease
vCJD: Variant Creutzfeldt–Jakob disease
MRI: Magnetic Resonance Imaging
EEG: Electroencephalography
DWI: Diffusion Weighted Imaging
SWI: Susceptibility Weighted Imaging
FLAIR: Fluid-attenuated inversion recovery
RT-QuIC: Real-time quaking-induced conversion
PSWCs: Periodic sharp wave complexes
PLED: Periodic lateralized epileptiform discharges
PMCA: Protein misfolding cyclic amplification
FIRDA: Frontal intermittent rhythmic delta activity
PrP: Prion protein
V: Valine
CDC: Centers for disease control and prevention
CSF: Cerebrospinal fluid
